# An Experimental Study on Hydrodynamic Retention of Low and High Molecular Weight Sulfonated Polyacrylamide Polymer

**DOI:** 10.3390/polym11091453

**Published:** 2019-09-05

**Authors:** Sameer Al-Hajri, Syed M. Mahmood, Ahmed Abdulrahman, Hesham Abdulelah, Saeed Akbari, Nabil Saraih

**Affiliations:** 1 Department of Petroleum Engineering, Universiti Teknologi PETRONAS, Seri Iskandar, Perak 32610, Malaysia; 2 College of Petroleum and Geoscience, King Fahd University of Petroleum and Minerals, Dhahran 34463, Saudi Arabia

**Keywords:** polymer flooding, polymer retention, hydrodynamic retention, sulfonated polyacrylamide

## Abstract

Polymers are often added with water as a viscosifier to improve oil recovery from hydrocarbon reservoirs. Polymer might be lost wholly or partially from the injected polymer solution by adsorption on the grain surfaces, mechanical entrapment in pores, and hydrodynamic retention in stagnant zones. Therefore, having a clear picture of polymer losses (and retention) is very important for designing a technically and economically successful polymer flood project. The polymer adsorption and mechanical entrapment are discussed more in depth in the literature, though the effect of hydrodynamic retention can be just as significant. This research investigates the effect of the hydrodynamic retention for low and high molecular weight (AN 113 VLM and AN 113 VHM) sulfonated polyacrylamide polymer. Two high permeability Bentheimer core plugs from outcrops were used to perform polymer corefloods. Polymer retention was first determined by injecting 1 cm^3^/min, followed by polymer core floods at 3, 5, and 8 cm^3^/min to determine the hydrodynamic retention (incremental retention). A higher molecular weight polymer (AN 113 VHM) showed higher polymer retention. In contrast, hydrodynamic retention for lower molecular weight (AN 113 VLM) was significantly higher than that of the higher molecular weight polymer. Other important observations were the reversibility of the hydrodynamic retention, no permanent permeability reduction, the shear thinning behavior in a rheometer, and shear thickening behavior in core floods.

## 1. Introduction

Polymer flooding is an enhanced oil recovery process in which a specific polymer concentration (usually 200–1500 ppm) is added to injected water. Polymers increase the viscosity of the injected solution and therefore preferably decrease the water to oil mobility ratio [1], which results in improving the displacement efficiency of the injected displacing solution [2,3,4]. However, significant interactions between the polymer and the porous media, causing the polymer molecules to be lost or retained in the porous media [5,6,7,8,9]. This results in the formation of a frontal bank denuded wholly or partially of the polymer.

In previous research [2,7,10,11,12,13,14,15,16], polymer retention during polymer solution flow through porous media was investigated. There are three polymer retention mechanisms: polymer adsorption, mechanical entrapment, and hydrodynamic retention [17]. Adsorption results from the interactions between polymer molecules and the rock-solid surface [15]. This interaction causes the molecules to bond to the surface of the solid mainly by physical adsorption, van der Waals forces, and hydrogen bonding. Moreover, mechanical entrapment and hydrodynamic retention can significantly contribute to polymer retention in low permeable and fluctuating flow rates in porous media [18,19].

Hydrodynamic retention is the least investigated and reported retention mechanism and is neglected in most polymer flooding applications [18]. Chauveteau and Kohler noticed that Partially Hydrolyzed Polyacrylamide (HPAM) polymer retention changed with the change in flow rates [20]. Their experiment demonstrated the effect of hydrodynamic retention by increasing the flow rate from 3 to 10.3 m^3^/days. This resulted in more losses of polymer in the porous media. As the flow rate was lowered again to 3 m^3^/days, the effluent polymer concentration rose to the input concentration. 

Scientists suggested two main mechanisms to describe the effect of flow rate on polymer retention during polymer flooding. The first mechanism assumes that molecules of the polymer are temporarily trapped in the porous media in stagnant zones because of applied hydrodynamic drag force. The polymer molecules are expected to flow back in the mainstream if the flow is reduced or stopped [18,20].

The second mechanism refers to the trapping of the polymer to the molecules shape. Polymer molecule shape will change from coiled to elongated and stretched molecules at higher shear rates in the restricted flow areas. The small size of the elongated molecules allows them to flow through pores of smaller sizes. Thus, more molecules will be trapped. At the extremely high shear rate, polymer retention increases because of the deformation of the elongated polymer molecules [21,22,23].

Polymer adsorption has been widely studied for Partially Hydrolyzed Polyacrylamide (HPAM) and Polyacrylamide (PAMS) with a specific sulfonation degree. Some studies in the literature suggested lower retention for sulfonated polyacrylamide polymers as compared to HPAM polymers [24,25,26], which made some of the sulfonated polymers better candidate viscosifiers for polymer flooding applications. 

The hydrodynamic retention for HPAM polymers was fairly reported in the literature [27,28]. So far, there is no study reported the hydrodynamic retention performance of the sulfonated polymers. This study focuses on the interaction between the sulfonated polyacrylamide polymers and rock surfaces of Bentheimer cores at various flow rates. The effect of molecular weight on polymer retention and hydrodynamic retention, reversibility of the sulfonated polyacrylamide polymers, permeability reduction, and rheological behavior of a higher molecular weight polymer (AN 113 VHM) and hydrodynamic retention for lower molecular weight (AN 113 VLM) were investigated.

## 2. Materials and Methods

### 2.1. Polymer and Brine

Sulfonated polyacrylamide polymers (AN 113 VLM and AN 113 VHM) were used in this study. Polymers were provided as white granular powders from SNF Floerger, Andrézieux, France. The molecular weight of AN 113 VLM is estimated of approximately 0.5–1 million Daltons with a sulfonation degree of 13% and AN 113 VHM of approximately 11–12 million Daltons with a sulfonation degree of 13%. The polymer solution was prepared using the magnetic stirrer vortex method. Ultra-pure water was used with 5000 ppm Sodium Chloride (NaCl) salinity. The rheology of AN113 VLM and AN 113VHM polymers were determined using TA Rheometer (Thermal Analysis and Rheology Instruments, TA Instruments, Inc., New Castle, DE, USA) at a shear rate ranges between (0.1–500 S^−1^), the viscosity measurement was conducted at the room temperature (25 °C).

### 2.2. Dynamic Adsorption Measurement

Bentheimer sandstone core samples have proven to be ideal for laboratory studies because of their block scale homogeneous nature and lateral continuity. They have been widely utilized to study reservoir issues ranging from passive and active properties of oil, gas, water, and rock interactions and processes to flow and transport. The physical properties of the two Bentheimer cores used for core flooding experiment are shown in Table 1. The porosity of the two cores was slightly similar to about 19.5%. In a similar fashion, B1 and B2 had a permeability of 1991 and 1932 mD respectively. The cores were evenly cut to yield a similar length to some extent to be able to compare the retention results.

Solutions of AN113 VLM and AN113 VHM powders are prepared by mixing and dissolution. Ultra-pure water was stirred in a beaker by a magnetic stirrer at 720 rpm and 5000 ppm NaCl was added to the ultra-pure water to make the base brine. Finally, dry polymer powder was slowly introduced (to avoid the formation of fish-eyes) to the brine. To ensure complete dissolution, the solution was stirred for 24 h. The compositions and concentrations of all samples prepared are shown in Table 2.

A similar base solution (having the same NaCl concentration) was prepared and vacuumed from the air using a desiccator. After the core was weighted in its dry state, it was saturated with the degassed base solution and vacuumed in the desiccator for 24 h to make sure almost all the pores in the core was saturated with brine. The core was then weighed again to determine its porosity and pore volume using the gravimetric analysis. 

**Figure 1:**
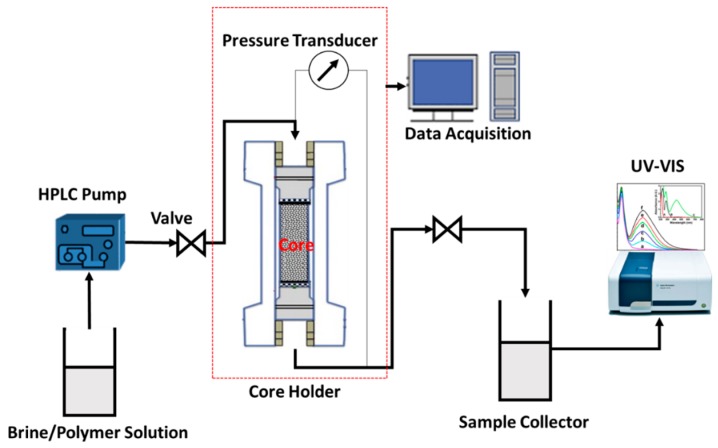
Schematic of the unit used for polymer retention determination.

Benchtop Permeability System (BPS) manufactured by Coretest Systems Corporation, Reno, NV, USA was used in performing the core flood for dynamic adsorption in the porous media. We used the extended injectivity method used by Lotsch and Law for the dynamic adsorption test [29]. In this method, two polymer solution slugs of the same composition are injected to the core and separated by an extended brine injection. Polymer retention can be determined from the area under the two curves of the polymer effluent normalized concentration plotted versus injected pore volume. A schematic of the unit used in the dynamic adsorption test is shown in Figure 1. 

Polymer-enhanced waterflooding experiments were carried out on two Bentheimer sandstone cores (permeabilities of 1991 and 1935 mD and porosity of 19.5%). AN 113 VLM and AN 113 VHM polymers of 1000 ppm concentrations were used with 5000 ppm NaCl at a fixed flow rate of 1 cm^3^/min (equivalent to the frontal velocity of 22 ft/day).

The clean dried cores were evacuated and pre-saturated with brine by placing them in a desiccator. To assure that any residual gas was removed, brine was injected until reaching the steady-state condition (required about 3 pore volume (PV) of brine injection).

The injection was switched from brine to polymer solution until about 2.5 PV of polymer slug injection, during which 4 cm^3^ of effluent samples were collected every four minutes for concentration analysis. The injection was switched back from polymer solution to brine injection in order to flush the reversible polymer that was not adsorbed or trapped in the porous media. The post-polymer brine injection continued till 80 PV of brine was injected.

Finally, the second slug (2.5 PV) injection of the same polymer solution was started during which the effluent samples were also collected just like during the first slug injection. Table 3 summarizes the steps followed during this coreflood test. 

The first test was performed using AN 113 VLM polymer. The same procedure was also followed for AN 113 VHM.

### 2.3. Hydrodynamic Retention Measurement

To investigate hydrodynamic retention, the cores were initially tested for polymer retention, as explained in the previous section. Pore volume (PV) of a core plug is obtained by multiplying its bulk volume with its porosity; it is often used as a measure of how much fluid has passed through the core. Then, 2.6 pore volume of the polymer solution was injected at different flow rates (3, 5, and 8 cm^3^/min) with an 80 PV of post-polymer injection. Samples were collected every 4 cm^3^ for each injected polymer slug. The polymer hydrodynamic retention was calculated from the area between the second polymer slug at a low flow rate (1 cm^3^/min) and the first high flow rate polymer slug.

### 2.4. Polymer Concentration Measurement

The UV-Vis method has proven to be trusted and convenient for measuring the concentration of polymers [30,31]. Agilent Cary 60 Spectrophotometer with a wavelength between 1100–190 nm (Agilent Technologies, Santa Clara, CA, USA) was used to determine the polymer concentration in both dynamic and static tests. Cary 60 (Agilent Technologies) was utilized to measure the absorbance of the polymer as a function of the wavelength (peaking at 330–300 nm) of the electromagnetic spectrum.

## 3. Results and Discussion

The results of this laboratory study on the retention, hydrodynamic retention and rheology of AN 113 VLM and VHM polymers are presented in the following section with the objective to see the role of molecular weight in polymer performance of polymer-enhanced waterflooding through porous media.

### 3.1. Retention Measurement

**Figure 2:**
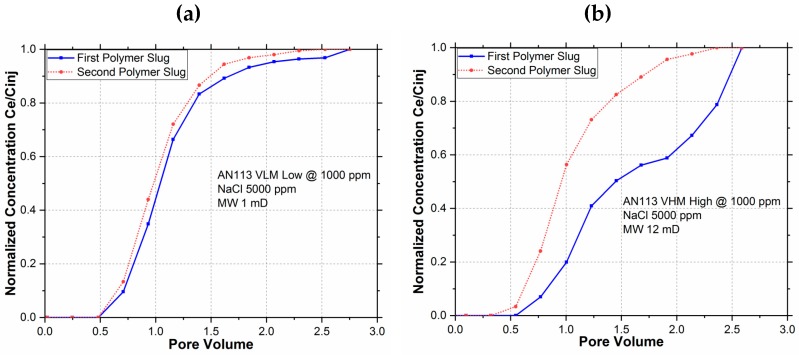
Breakout curves for 1000 ppm polymer concentration and 5000 PPM NaCl salinity for: (**a**) AN113 VLM (M_W_ = 1 mD) and (**b**) AN113 VHM (12 mD).

Figure 2 shows the normalized concentration of the injected and effluent polymer solution plotted versus the pore volume injected. These breakout curves are used to estimate polymer retention by finding the area between the two polymer slug injection curves. OriginPro® software (by OriginLab, Northampton, MA, USA) was used to estimate the area between the curves, which was then used to calculate retention in microgram of polymer per gram of rock.

Although the second polymer slug curves for both polymers are quite similar, the first slug curves show a significant deviation, which points out that the molecular weight does play a significant role in adsorption behavior.

**Figure 3:**
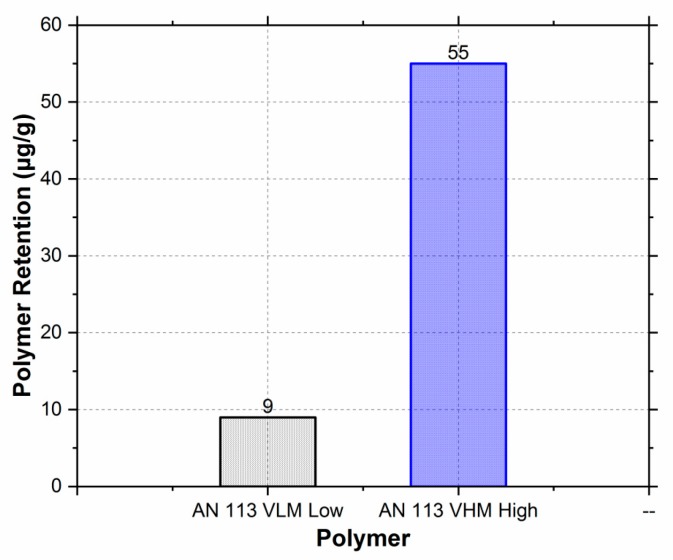
Retention behavior at 1000 ppm of polymer with 5000 ppm NaCl salinity.

Figure 3 shows the polymer retention for the two polymers as a bar chart. The retention was significantly higher (55 µg/g) for the higher molecular weight polymer (AN113 VHM) as compared to the lower molecular weight polymer (AN113 VLM).

The exact reason for the difference in retention capacity between the high and low molecular weight polymers is unknown. It could be due to mechanical entrapment or because of the molecular weight difference itself since the polymer retention is reported as the mass of the polymer.

### 3.2. Effect of Rate on Polymer Retention

Polymer retention experiments were performed at four different flow rates using AN 113 VLM (low M_W_) and AN 113 VHM (high M_W_) polymers. The effluent absorbance was detected at 300–320 nm wavelength and converted to polymer concentration using the laboratory determined calibration curves. Polymer retention was determined from the area between the breakout curves of first and second polymer slugs.

**Figure 4:**
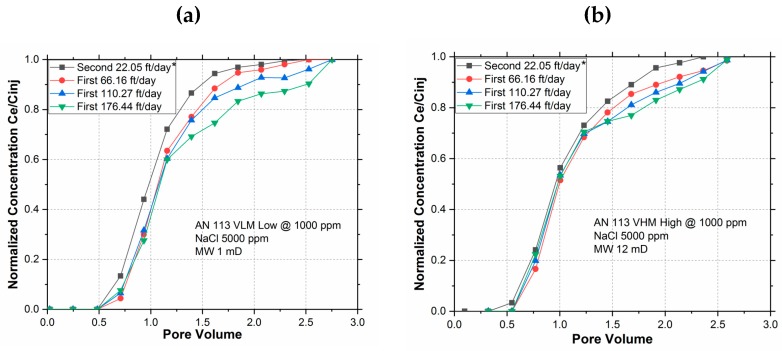
Polymer breakout curves at various rates for (**a**) AN 113 VLM and (**b**) AN 113 VHM. *The “Second 22 ft/day” breakout curve represents no adsorption case, hence is usable as the reference slug to determine the area between the curves for all runs of different flow rates.

Figure 4 shows the hydrodynamic retention of the two polymers at different flow rates (1, 3, 5, and 8 cm^3^/min) corresponding to frontal velocities of 22, 66, 110, and 176 ft/day. Table 4 lists the hydrodynamic retention results of both polymers.

**Figure 5:**
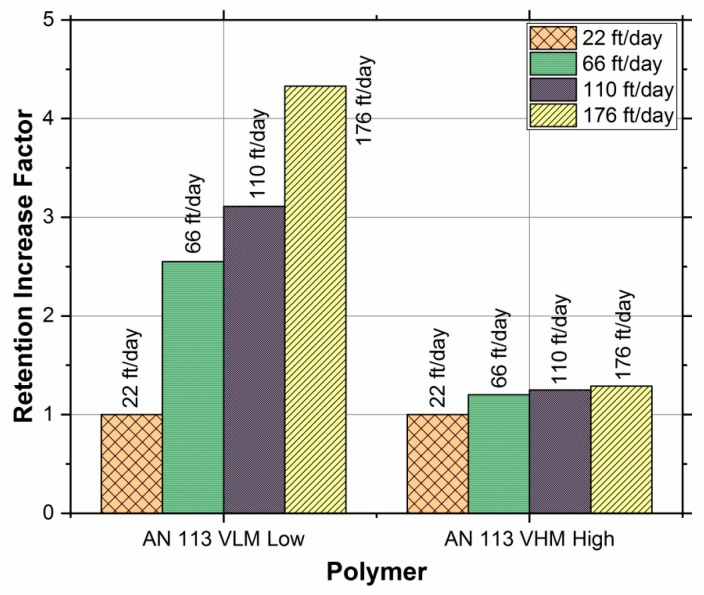
Retention Increase Factor at various rates for AN 113 VLM and AN 113 VHM.

Polymer retention increased with growing interstitial velocity for both polymers due to hydrodynamic retention (HDR), as expected. However, the HDR and its sensitivity to injection rate were both significantly greater for lower M_W_ polymer (AN 113 VLM) when compared to the higher M_W_ polymer (AN 113 VHM), as shown in the bar chart Figure 5. The adsorption is generally considered to be the dominant retention mechanism [20,30] and the results of high M_W_ polymer are consistent with this consensus. In case of a low M_W_ polymer, however, the HDR was much more significant than the adsorption. This observation is important for selecting the M_W_ of polymer in reservoirs, where the velocity is the highest near the wellbore.

The exact reason for the significant increase in HDR of low M_W_ as compared to the one with high M_W_ polymer is not known. However, this observation might be explained by the fact that the physical volume of the solvated polymer molecule is higher for higher molecular weight polymers; thus, the number of molecules retained in a given stagnant zone will be less for higher molecular weight polymer. Another possible reason could be the osmotic pressure, which is the thermodynamic driving force for mixing. For large molecules, this force is relatively small as compared to the viscous forces which are proportional to the polymer injection rate.

The increase in hydrodynamic retention for lower M_W_ polymer is suggesting that more polymer molecules will reside in a stagnant zone if their size is small. However, the results are different than the once which were observed by Chen et al. [27]. The difference can be explained on the basis of the order of magnitude difference in permeability of the cores in the two studies. Chen’s study used a low permeability core (167 mD), which encounters a greater retention contribution from mechanical entrapment. This study, on the other hand, used a high permeability core (2000 mD) in which the chances of mechanical entrapment are greatly reduced.

**Figure 6:**
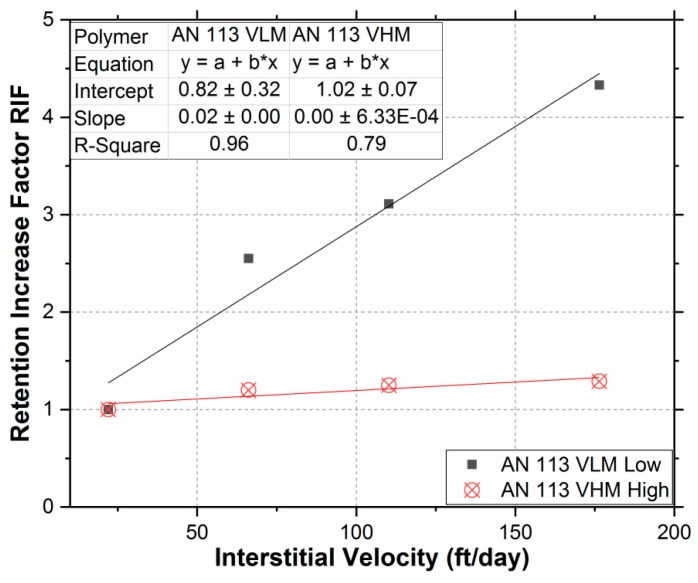
The increase in polymer retention factor with interstitial velocity.

To see if there is a correlation between polymer retention and interstitial velocity, the data in Table 4 was plotted in Figure 6. A good correlation between polymer retention increase factor (RIF) and interstitial velocity was observed. For both low and high molecular weights, the hydrodynamic retention increased almost linearly with increasing interstitial velocity within the range of velocities tested and are likely to be encountered at field conditions. The velocity effect was more pronounced in AN 113 VLM (low molecular weight) polymer than AN 113 VHM for the reasons discussed earlier.

### 3.3. Reversibility of Hydrodynamic Retention

It is generally understood that HDR is a reversible phenomenon [28]. However, the role of MW on the reversibility of HDR in sulfonated polyacrylamide polymers has not been previously published. Two polymer core floods were performed using AN 113 VLM and AN 113 VHM to investigate this role.

Before the start of the HDR tests, it was presumed that the cores had already completed adsorption as a result of previous tests. The cores were flushed out with 80 PV of brine injection. Then the first polymer slug was injected at 176.44 ft/day; 4 cm^3^ of effluent samples were collected frequently. The reversible polymers were flushed out with 80 PV of brine injection. The second polymer slug was then injected at the same rate of 176.44 ft/day and effluent samples were collected.

**Figure 7:**
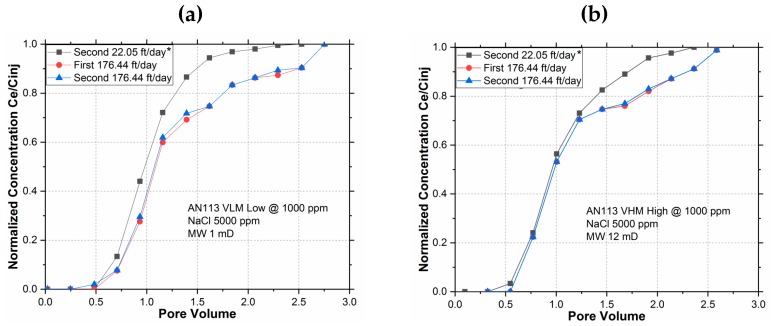
Polymer Hydrodynamic Retention Reversibility for (**a**) AN 113 VHM and (**b**) AN 113 VHM. * The “Second 22 ft/day” breakout curve was determined in earlier experiments. It represents no adsorption case, hence is usable as the reference slug to determine the area between the curves for all subsequent breakout curves including the first and second 176 ft/day breakout curves shown in this figure.

The breakout curves are shown in Figure 7. In addition to the two 176.44 ft/day polymer injection breakout curves, the “Second 22 ft/day” breakout curve is also included. This curve was determined in an earlier experiment during the adsorption-free slug injection run; it is used in Figure 7 as the reference slug for determining the adsorption during each of the two 176.44 ft/day polymer slug injection.

Since neither of the breakout curves at a high interstitial velocity of 176.44 ft/day approached (matched) the adsorption-free “Second 22 ft/day” reference breakout curve, it is evident that extra HDR was encountered in both. Moreover, the close match between the first and the second 176 ft/day breakout curve indicates that the HDR did not change during the subsequent identical polymer injection. This is only possible if the HDR during the first 176 ft/day polymer injection was completely reversed by the 80 PV of washout brine injection before the second 176 ft/day polymer injection. Since the reversibility of HDR was observed in both low and high M_W_ polymers, it could be surmised that the MW did not have any effect on HDR.

### 3.4. Permeability Reduction

Differential pressures were noted during pre and post polymer brine injection at 22 ft/day and the Residual Resistance Factor (RRF) was calculated as follows:

RRF = ΔP_post_/ΔP_pre_ (1)

where ΔP_pre_ is the differential pressure (0.018 psi for AN 113 VLM and 0.02 psi for AN 113 VHM) during brine injection before polymer introduction and ΔP_post_ is the differential pressure during brine injection after the core had been flooded with polymer at various rates.

The RRF is an effective indicator of permeability reduction caused by the polymer retention and also indicates its reversibility. Table 5 shows the values of RFF along with the differential pressures during brine injection after each polymer slug injection.

**Figure 8:**
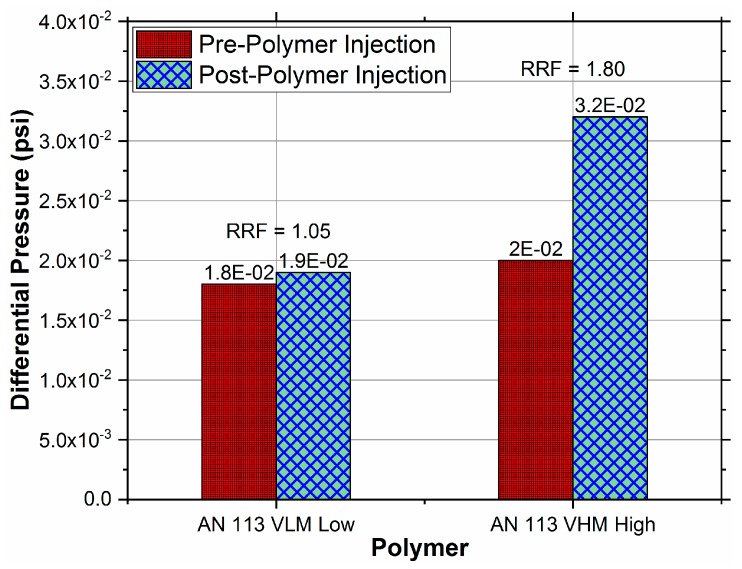
Differential pressures and Residual Resistance Factor (RRF)for pre- and post-polymer injection.

The data in Table 5 (also presented in Figure 8 as a bar graph) shows that the adsorption is not completely reversible since even after injecting significantly large volumes (80 PV) of brine, the ΔP during brine injection at the identical rate did not revert to the pre-polymer condition. This resulted in an RRF higher than unity (1.05, 1.80) for polymers of both molecular weights. This shows that adsorption causes irreversible permeability reduction.

The fact that the pressure drop during brine injection, intended to flush out the polymer, always reached the same level (0.019 or 0.032 psi) indicates that the brine was able to completely flush out all hydrodynamically retained polymer even though the polymer slugs prior to the brine flush were injected at variable rates (22, 66, 110, 176 ft/D) for various tests.

It is noteworthy to mention that the RRF value determined for AN 113 VLM (low M_W_) was lower than RRF value for AN 113 VHM (high M_W_), indicating a higher permeability reduction for polymers with higher molecular weights. However, the hydrodynamic retention in both polymers did not alter permeability permanently and was completely reversible regardless of the M_W_.

### 3.5. Rheological Behaviour of AN 113 VLM and AN 113 VHM

The rheological behavior of the polymers was investigated by bulk methods as well as in situ behavior through coreflood experiments, in order to compare different methods and the effect of M_W_ as described in the following sections.

#### 3.5.1. Bulk Rheology Using Rheometer

**Figure 9:**
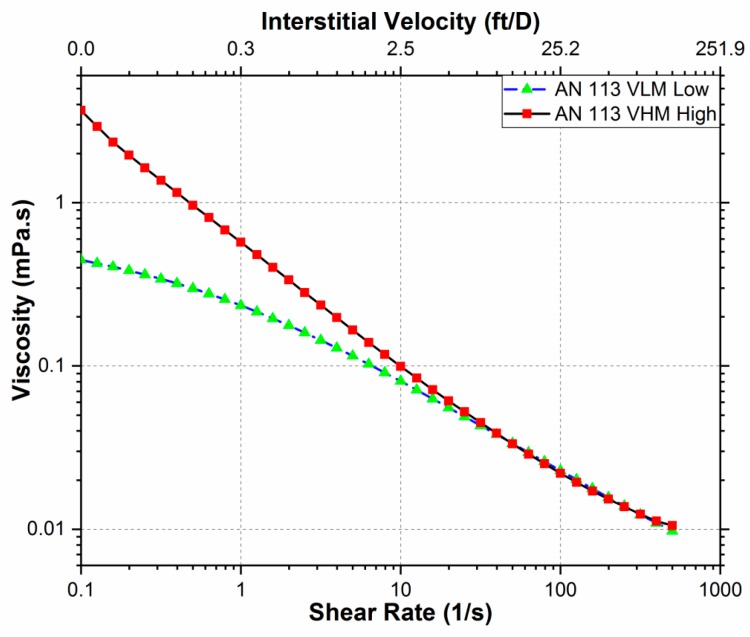
The bulk rheology of AN 113 VLM and AN 113 VHM at a concentration of 1000 ppm and salinity of 5000 ppm.

Figure 9 shows data from the rheological study of 1000 ppm concentration with 5000 ppm salinity of the two polymers. The polymer solutions exhibited shear thinning behavior and a significant decrease in polymer solution viscosity (40 and 400 times) in the range of shear rates tested (0.1–500 s^−1^). The shear rate measured using the rheometer was then converted to velocity using Equation (2) [32]:

(2) $$\gamma =4\alpha v\sqrt{\frac{\Phi }{8k}}$$

where γ is the shear rate in s^-1^, α is a dimensionless tuning parameter associated with the rock particles (2.5), v is the interstitial velocity in m/s, Φ and k are the porosity and permeability of the core.

This behavior was expected since most polymer solutions exhibit shear thinning because of the decrease in the hydrodynamic size of the polymer [33,34]. Increasing shear rate causes greater breakage and destruction of the entanglements between the polymer molecules, thereby reducing polymer viscosity [35,36]. An increase in shear rate may also result in higher retention as more porous media will be exposed to the polymer solution due to the reduced hydrodynamic size of the polymer.

#### 3.5.2. In Situ Rheology Using Core Flood

To investigate the rheology of the polymer in porous media, Resistance Factors (RF) were determined during the injection of each polymer slugs at various flow rates. Resistance factor is defined here as the ratio pressure drop during polymer injection to the pressure drop during brine injection (ΔP_polymer_/ΔP_brine_). Table 6 shows the pressure differential drops across the two cores at various flux rates and corresponding RF’s.

**Figure 10:**
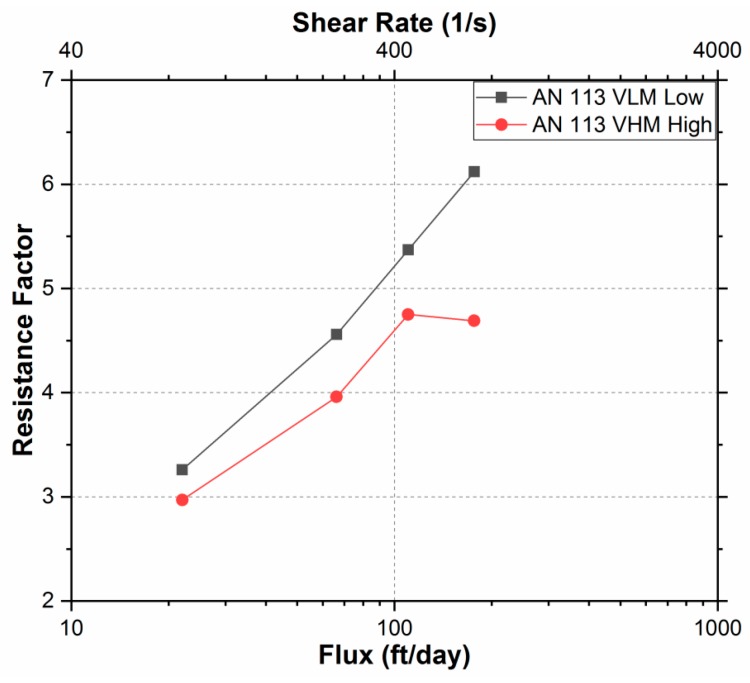
Rheological Behavior in porous media of AN113 VLM and AN113VHM polymers.

As compared to AN 113 VHM, the lower molecular polymer (AN 113 VLM) had higher RF for any given rate. A higher resistance indicates that more polymer molecules were retained in porous media. This observation agrees with the results obtained in the previous section that the lower MW polymer encounters higher hydrodynamic retention. Figure 10 illustrates the type of flow behavior for the two polymers in the same range of flux.

AN 113 VLM showed a shear thickening behavior (slope = 3.2) with a high dependency of resistance factor to flux rate. AN 113 VHM also showed a shear thickening behavior in the flux range of 22 to 110 ft/day (slope = 2.5); however, the resistance factor decreased at an increased rate of 176 ft/day, showing shear thinning behavior. This decrease in RF possibly results from viscosity reduction caused by mechanical degradation at higher velocities [37,38]. 

The shear thickening behavior of the two sulfonated polyacrylamide polymers in porous media is due to the viscoelasticity of polymer molecules and elongational flow field [28,39]. At high velocities, polymer molecules remain in a coiled state because time is not sufficient for the molecules to become stretched before they flow through a pore constriction. The coiled polymer molecules show higher resistance to flow than stretched ones. Therefore, greater force or pressure gradient is required to force the coiled molecules through pore throats, resulting in higher resistance factor as the velocity increases [28]. This shows that the shear thickening behavior of the sulfonated polyacrylamide polymer was an intrinsic property resulting from the viscoelasticity of polymer molecules and elongational flow field.

## 4. Conclusions

This study focused on the interaction between the sulfonated polyacrylamide polymers and rock surfaces of Bentheimer cores at various flow rates. The effect of molecular weight on polymer retention and hydrodynamic retention was also investigated. The reversibility, permeability reduction, and rheological behavior of AN 113 VLM and AN 113 VHM were also investigated in this study. The following conclusions were made:

- Polymer retention increased when the molecular weight of the AN 113 sulfonated polyacrylamide polymer also increased.
- The polymer retention (adsorption) of AN 113 VLM and AN 113 VHM caused irreversible permeability reduction, which increased the residual resistance factor of both (1.05 and 1.80).
- For unclear reasons, hydrodynamic retention was noticeably higher for lower molecular weight polymers.
- The polymer hydrodynamic retention was reversible and had no permanent effect on permeability reduction.
- Both AN 113 VLM and AN 113 VHM sulfonated polyacrylamide polymers showed shear thinning behavior in the bulk rheological study using Rheometer.
- The core flooding study showed that the resistance factor increased when the flow rate increased, which resulted in a shear thickening behavior for AN 113 VLM and AN 113 VHM. However, at very high flow rates, mechanical degradation may cause shear thinning behavior.

Since the flow from the injection towards the production well is radial, the flow rates are very high near the wellbore and gradually reduce as the flow cross-sectional area increases away from the wellbore. Therefore, in a polymer flooding enhanced oil recovery project, significant hydrodynamic retention could be anticipated near the wellbore due to high flow rates, which could dilute the polymer slug, rendering it ineffective. This study shows that the molecular weight should be taken into consideration while designing an optimum polymer slug since the hydrodynamic retention for lower molecular weight polymer was significantly higher than the retention for the higher molecular weight polymer.

It is advisable to perform dynamic polymer studies under reservoir conditions to screen polymers that are thermally and physically stable, in order to understand various mechanisms of polymer loss in porous media before selecting a suitable polymer.

## Figures and Tables

**Table 1:** Core Sample Properties.

Core ID	Lithology	Length (cm)	Weight (g)	Porosity (%)	Permeability (md)	Pore Volume (cc)
B1	Sandstone	7.6	169	19.5	1991	17.2
B2	Sandstone	7.6	170	19.2	1932	17.1

**Table 2:** Polymer solution composition.

Sample ID	Polymer Type	Concentration (ppm)	Molecular Weight (md)	Salinity (ppm)
P1	AN113 VLM	1000	0.5–1	5000
P2	AN113 VHM	1000	11–12	5000

**Table 3:** Summary of a higher molecular weight polymer (AN 113 VHM) and hydrodynamic retention for lower molecular weight (AN 113 VLM) polymer-enhanced water-floods.

Process	Injected PV *	Samples Collected
Pre-injection Brine	3	None
First Polymer slug	2.5	Every 4 cm^3^
Post-injection Brine	80	None
Second Polymer slug	2.5	Every 4 cm^3^

* All injection was carried out at a rate of 1 cm^3^/min which is equivalent to an interstitial velocity of 22 ft/day through the core. PV: pore volume.

**Table 4:** Hydrodynamic Retention for AN 113 VLM and AN 113 VHM.

Polymer	Polymer Injection Rate (cm^3^/min)	Interstitial Velocity (ft/Day)	Retention at base velocity of 22 ft/Day (µg/g)	Total Retention (µg/g)	Retention Increase Factor
AN113 VLM	1	22*	9	9	1.00
	3	66		23	2.55
	5	110		28	3.11
	8	176		39	4.33
AN113 VHM	1	22 *	55	55	1.00
	3	66		66	1.20
	5	110		69	1.25
	8	176		71	1.29

The interstitial velocity of 22 ft/day chosen from literature and used for normalization because it represents polymer retention with negligible influence of hydrodynamic retention.

**Table 5:** Residual Resistance Factor for AN 113 VLM and AN 113 VHM.

Parameter	AN 113 VLM	AN 113 VHM
Interstitial velocity during post-polymer brine injection for each test, ft/D	22	22
Initial ΔP during brine injection prior to any polymer injection, psi	0.018	0.020
ΔP post-polymer injection, psi *	0.019	0.032
Calculated RRF (ΔPpost/ΔPpre)	1.05	1.80

* Whereas the polymer was injected at 22, 66, 110, 176 ft/D for various tests, the brine injection for post polymer flood was always conducted at 22 ft/Day.

**Table 6:** Resistance Factor at Different Flux for AN 113 VLM and AN 113 VHM.

Polymer	Flux (ft/Day)	Shear Rate (1/s)	ΔPpolymer (psi)	Calc ΔP * brine (psi)	Calc RF (ΔP_polymer_/ΔP_brine_)
AN 113 VLM	22	87	0.062	0.019	3.26
	66	262	0.260	0.057	4.56
	110	436	0.510	0.095	5.37
	176	698	0.930	0.152	6.12
AN 113 VHM	22	87	0.095	0.032	2.97
	66	262	0.380	0.096	3.96
	110	436	0.760	0.160	4.75
	176	698	1.200	0.256	4.69
